# The prognostic significance of topoisomerase II alpha protein in early stage luminal breast cancer

**DOI:** 10.1186/s12885-018-4170-7

**Published:** 2018-03-27

**Authors:** Xin An, Fei Xu, Rongzhen Luo, Qiufan Zheng, Jiabin Lu, Yanhua Yang, Tao Qin, Zhongyu Yuan, Yanxia Shi, Wenqi Jiang, Shusen Wang

**Affiliations:** 1 State Key Laboratory of Oncology in South China; Collaborative Innovation Center for Cancer Medicine, 651 Dongfeng Road East, Guangzhou, People’s Republic of China; 2 Department of Medical Oncology, Sun Yat-sen University Cancer Center, 651 Dongfeng Road East, Guangzhou, People’s Republic of China; 3 Department of Pathology, Sun Yat-sen University Cancer Center, 651 Dongfeng Road East, Guangzhou, People’s Republic of China

**Keywords:** Luminal breast cancer, Late recurrence, Prognostic factor, Survival, Topoisomerase II alpha

## Abstract

**Background:**

Topoisomerase II alpha (TOP2A) protein has been shown to be a proliferation marker associated with tumor grade and Ki67 index. The prognostic effect of TOP2A seems different among different subtypes of breast cancer. The current study evaluated the prognostic impact of TOP2A protein on luminal breast cancer.

**Method:**

Altogether 434 stage I-II luminal breast cancer patients who underwent curative surgery in Sun Yat-Sen University Cancer Center between 2007 and 2009 were enrolled. TOP2A protein expression was assessed by immunohistochemistry. Clinical and pathological data were retrospectively collected.

**Result:**

With a cut-off value of 30%, 127 (29.3%) patients were classified as TOP2A overexpression. TOP2A overexpression was associated with a higher tumor grade and Ki67 index. Patients with TOP2A high expression showed a significantly higher rate of distant metastasis and shorter distant metastasis free survival (DMFS) compared with patients with low TOP2A expression. The prognostic influence of TOP2A expression was more significant in years 5–8 after diagnosis, and more pronounced in stage II patients, luminal B disease, and patients treated with adjuvant endocrine therapy alone. Multivariate survival analysis revealed TOP2A overexpression was an independent fact for worse DMFS.

**Conclusion:**

TOP2A protein showed a time dependent influence on prognosis in stage I-II luminal breast cancer, suggesting it might be a potential predictor of late recurrence for this group of patients.

## Background

Luminal breast cancer, defined by the presence of estrogen receptor(ER) and/or progesterone receptor (PgR) and absence of human epidermal growth factor receptor 2 (HER2), is the most common subtype of breast cancer. This subtype generally shows favorable survival, better response to endocrine therapy, and less benefit from chemotherapy [1, 2]. Role of adjuvant chemotherapy is controversial in localized luminal breast cancer, especially for those at early stage. It is well recognized that luminal breast cancer is a heterogeneous group of tumors with a large variation in prognosis and sensitivities to treatment [3, 4]. Although the majority of patients are good candidates for endocrine therapy, there is sub-population who show resistance to endocrine therapy and would benefit from chemotherapy [5, 6]. Thus, it is crucial to identify this sub-population properly.

Extended gene expression profiling (GEP) has divided luminal breast cancer into A and B subtypes that improves prognostication and prediction of response to therapy [1, 7]. However, the best method to define molecular classification of breast cancer is still a major clinical issue. The “gold standard” GEP assay requires fresh-frozen tissues and complicated technique, therefore, is usually not feasible [1]. Quantitative reverse-transcriptase polymerase chain reaction (RT-qPCR) based multigene assays such as Oncotype DX and Mammaprint are efficient and commercially available; however, these assays are expensive, and have not been prospectively validated [8, 9]. The widely used immunohistochemistry (IHC) surrogate approach is cheap and simple, but shows low consistency in classifying Luminal A and B subtypes. One of the main challenges for the IHC surrogate classification is to assess the proliferative activity of tumor cells correctly. Currently, Ki67 is the only proliferation marker recommended by St. Gallen consensus to distinct luminal A and B tumors [10]; however, it often exhibits staining heterogeneity. Also, assessment methods among different laboratories vary widely. Although different cutoff points of 14% [10] and 20% [11] were proposed, there was still large inconsistence about the value of Ki67 as a single marker as well as the best threshold to distinguish luminal A and B diseases [12]. Therefore, identifying additional biomarkers besides Ki67 is quite necessary to better stratify luminal breast cancer for individualized treatment.

Topoisomerase II alpha (TOP2A) is encoded by the *TOP2A* gene located on chromosome 17q12-q21. It is a key nuclear enzyme for controlling of topological states of DNA by generating transient breakage in double-stranded DNA; therefore it is involved in processes such as DNA replication and transcription, and chromosome formation, enrichment, and separation [13]. Abnormality of TOP2A plays a critical role in chromosome instability and tumorigenesis [14]. TOP2A is also reported to be the direct target of anthracyclines to cause DNA damage [15]. Like Ki67, TOP2A is regarded as a proliferation marker which is strongly expressed in proliferating cells [16, 17]. Expression of TOP2A was higher in proliferative subtypes of breast cancers such as triple negative and HER2-enriched diseases than luminal type [18]. However, high expression of TOP2A protein seemed to be associated with poor prognosis only in luminal breast cancer. Peter Fritz et al. enrolled 225 un-subtyped breast cancer patients and found the prognostic impact of TOP2A protein was only confined to hormone receptor positive patients [19]. Another two studies consistently reported that TOP2A mRNA expression was highly prognostic only in luminal type breast cancer [20, 21].

The current study enrolled a relatively large group of homogenously early luminal breast cancer patients (stage I-II), and try to confirm the prognostic value of TOP2A protein on this subtype of breast cancer classified by current IHC approach.

## Methods

### Patient population

Clinicopathological data on patients who referred for breast cancer surgery to Sun Yat-Sen University Cancer Center between January 2007 and December 2009 were retrospectively retrieved. As a result, 434 pathologically confirmed stage I-II, hormone receptor positive, HER2-negative breast cancer patients with complete data and available primary tumor samples were enrolled. The stage of disease was re-defined according to the American Joint Committee on Cancer stage system (AJCC) for breast cancer 7th Edition (2010). Hormone receptor positive was defined as ER and/or PgR positive ≥10% by IHC; HER2 negative was defined as IHC 0–1+, or IHC 2+, FISH negative [22]. Patients were further categorized into either Luminal A or B subgroup based on IHC-based surrogate definitions according to St. Gallen Consensus 2013**:** luminal A: ER and PgR positive, HER2 negative and Ki-67 “low” (< 20%); luminal B (HER2−): ER positive, HER2 negative and at least one of: Ki-67 “high” (≥20%) or PgR negative [11].

### TOP2A protein expression

TOP2A IHC staining was performed on formalin-fixed paraffin-embedded tumor samples using an automatic immunostainer (BenchMark XT; Ventana Medical Systems, Tucson, Ariz) according to the manufacturer’s instructions. The primary antibody used was clone Ki-S1 (Gene Tech, Shanghai) at a dilution of 1:100. All the specimens were examined and scored by two independent pathologists without the knowledge of patients’ data. Only nuclear staining (the active isoform of TOP2A) was considered. For each sample, 5 microscopic fields at × 200 magnification were selected, and 100 tumor cells in each field were counted to assess the staining intensity (0, 1+, 2+, 3+) and percentage of positive cells. The average positive percent rate was calculated as the final result. TOP2A protein overexpression was defined as ≥30% positive cells.

### Statistical analysis

The primary endpoints were disease-free survival (DFS) which was further divided into distant metastasis-free survival (DMFS) and locoregional recurrence-free survival (LRFS), breast cancer specific survival (BCSS), and overall survival (OS). Survival curves were plotted by the Kaplan–Meier method and compared by the log-rank test. Multivariate Cox regression analysis was performed to identify independent variables for survival. Associations between TOP2A expression and clinicopathological characteristics were assessed by the chi-square test (category variables), or the two-sample t test (continuous variables). All statistical tests were two-sided with *P* < 0.05 was considered statistically significant.

## Result

### Patient characteristics

Clinicopathological characteristics of all patients were summarized in Table 1. All patients were women. Patients in pathologic stage T1N0M0 (I), T1 N1/T2N0M0 (IIA), and T2 N1/T3N0M0 (IIB) were 131 (30.2%), 194 (44.7%), and 109 (25.1%). No patients received neoadjuvant therapy. Adjuvant chemotherapy was given to 384 (88.5%) patients. Radiotherapy was given to patients who had breast conserving surgery or tumor diameter greater than 5 cm, or those with 1–3 lymphatic metastases concomitant with other high risk factors for local recurrence. All patients received adjuvant endocrine therapy.

**Table 1:** Comparison of baseline characteristics between patients with high and low TOP2A protein expression

	All patients	TOP2A protein expression		
		High	Low	*P* value
	*n* = 434 (100%)	*n* = 127 (29.3%)	*n* = 307 (70.7%)	
Median age (range) a	45(26–80)	45(26–80)	45(26–80)	0.869
Age at surgery (yr)				0.725
≤ 40	124(28.6)	37 (29.1)	87 (28.3)	
> 40,< 60	288 (66.3)	82 (64.6)	206 (67.1)	
> =65	22 (5.1)	8 (6.3)	14 (4.6)	
Menopausal status				1.000
Premenopausal	323 (74.4)	95 (74.8)	228 (74.3)	
Postmenopausal	111 (25.6)	32 (25.2)	79 (25.7)	
Breast surgery				0.847
Lumpectomy	35 (8.1)	11 (8.7)	24 (7.8)	
Mastectomy	399 (91.9)	116 (91.3)	283 (92.2)	
Pathologic tumor size (mm)				0.244
≤ 20	176 (40.5)	57 (44.9)	119 (38.8)	
21–50	249 (57.4)	69 (54.3)	180 (58.6)	
> 50	9 (2.1)	1 (0.8)	8 (2.6)	
Number of involved lymph nodes				0.920
0	291 (67.1)	87 (68.5)	204 (66.5)	
1	77 (17.7)	22 (17.3)	55 (17.9)	
2	33 (7.6)	10 (7.9)	23 (7.5)	
3	33 (7.6)	8 (6.3)	25 (8.1)	
Predominant histologic subtype				0.765
Ductal	407 (93.8)	118(92.9)	289(94.1)	
Lobular	13 (3.0)	5 (3.9)	8 (2.6)	
Other	14 (3.2)	4 (3.2)	10 (3.3)	
Grade				0.028
1–2	304 (70.0)	79 (62.2)	225 (73.3)	
3	130 (30.0)	48 (37.8)	82 (26.7)	
Median Ki67 (range)^b^	10(2–90)	20(5–90)	10 (2–80)	0.018
Ki67				0.119
< 14%	220 (50.7)	59 (46.5)	161 (52.4)	
≥ 14%, < 20%	14 (3.2)	4 (3.1)	10 (3.3)	
≥ 20%,< 30%	37 (8.5)	7 (5.5)	30 (9.8)	
≥ 30%	163 (37.6)	57 (44.9)	106 (34.5)	
Adjuvant CT				0.869
No	50 (11.5)	16 (12.6)	34 (11.1)	
Anthracycline^c^	203 (46.3)	58 (45.7)	145 (47.2)	
Taxane^d^	9 (2.1)	2 (1.6)	7 (2.3)	
Anthracycline + Taxane^e^	172 (40.1)	51 (40.1)	121 (39.4)	
Adjuvant RT				1.000
Yes	81 (18.7)	24 (18.9)	57 (18.6)	
No	353 (81.3)	103 (81.1)	250 (81.4)	
Adjuvant ET				0.877
Tamoxifen/Toremifene	395 (91.0)	115 (90.6)	280 (91.2)	
AIs	15 (3.5)	6 (4.7)	9 (2.9)	
Tamoxifen/AIs	24 (5.5)	6 (4.7)	18 (5.9)	
Ovarian function suppression				1.000
Yes	14 (3.2)	4 (3.1)	10 (3.3)	
No	420 (96.8)	123 (96.9)	297 (96.7)	

*Abbreviations: CT* Chemotherapy, *ET* Endocrine therapy, *RT* Radiation, *AIs* Aromatase inhibitors

^a,b^Data were presented as number (range)

^c^including: EC regimen in 38 patients, FEC regimen in 165 patients

^d^including: TC regimen in 9 patients

^e^including: EC followed by docetaxel or paclitaxel in 16 patients, FEC followed by docetaxel or paclitaxel in 17 patients, TEC regimen in 10 patients, TE regimen in 129 patients

### TOP2A protein expression and correlation with clinicopathological characteristics

**Fig. 1:**
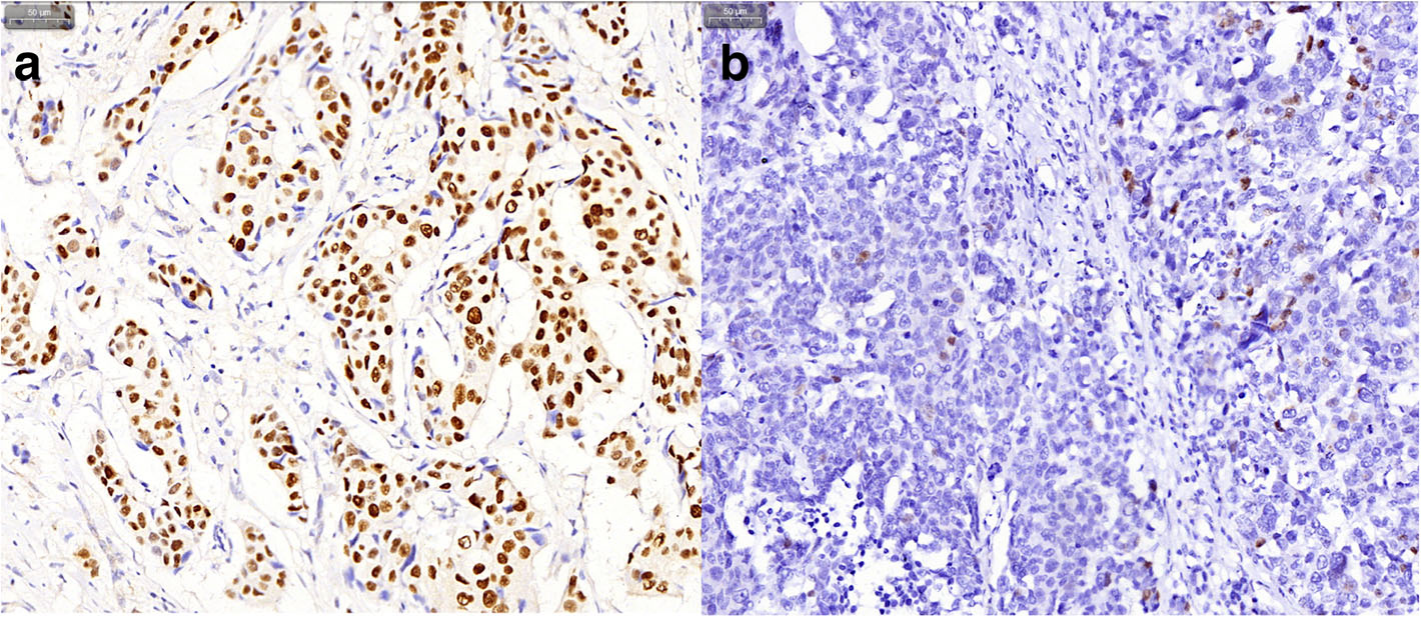
Representative immunostaining of TOP2A in luminal breast cancers. **a** High expression showing strong and diffuse nuclear staining of TOP2A (≥30%); (**b**) Low expression showing focal weak nuclear staining of TOP2A (< 30%) (magnification × 200 in each picture)

According to IHC assay, the nuclear staining intensity of TOP2A showed a significant positive correlation with the percentage of positive cells (*r* = 0.315, *P* < 0.001). Based on the cut-off value of 30%, 127 (29.3%) patients were classified as TOP2A overexpression, and 308 (70.7%) as no or low TOP2A expression. (Fig. 1).

The associations between TOP2A protein expression and clinicopathological characteristics were evaluated (Table 1). TOP2A overexpression was associated with a high tumor grade (*P* = 0.028). Median Ki67 index was also significantly higher in TOP2A overexpression group compared with that in TOP2A low group (*P* = 0.018). No association between TOP2A expression and age, menopausal status, tumor size, lymph node status, pathological subtypes, treatment modalities were found.

### Association between TOP2A protein expression and clinical outcome

The median follow up time was 80 months. Altogether 47 patients experienced recurrence of disease. Of these, nine patients experienced locoregional recurrence, 36 had distant metastases, and two patients had both locoregional and distant recurrence. A total of 21 patients died of breast cancer, and one patient died of heart failure. The 5-year DFS, DMFS, LRFS, BCSS and OS were 91.7, 93.5, 97.7, 96.2, and 96.0%; whereas the 8-year DFS, DMFS, LRFS, BCSS and OS were 86.9, 89.2, 97.0, 93.9, and 93.6%.

Overexpression of TOP2A was associated with significantly higher rates of all recurrence and distant metastasis, but not with locoregional recurrence. Patients with TOP2A high expression showed a significant higher rate of recurrence for years 5–8 compared with that in patients with low TOP2A expression. No significant difference of recurrence for years 0–5 was observed between the two groups. (Table 2).

**Table 2:** Number of recurrence for patients with high or low TOP2A expression according to site and time of recurrence

	All patients	TOP2A protein expression		
		High	Low	*P* value
	*n* = 434	*n* = 127	*n* = 307	
All recurrence n (%)	47(10.8)	21(16.5)	26 (8.5)	0.018
Site of recurrence^a^ n (%)				
Distant	38(8.8)	18 (14.2)	20 (6.5)	0.015
Locoregional	11 (2.5)	4 (3.1)	7 (2.3)	0.737
Time of recurrence n (%)				
0–5	31 (7.1)	12 (9.4)	17 (6.2)	0.213
5–8	16 (3.7)	9 (7.1)	7(2.3)	0.021

^a^There were two patients had both locoregional and distant recurrence

**Fig. 2:**
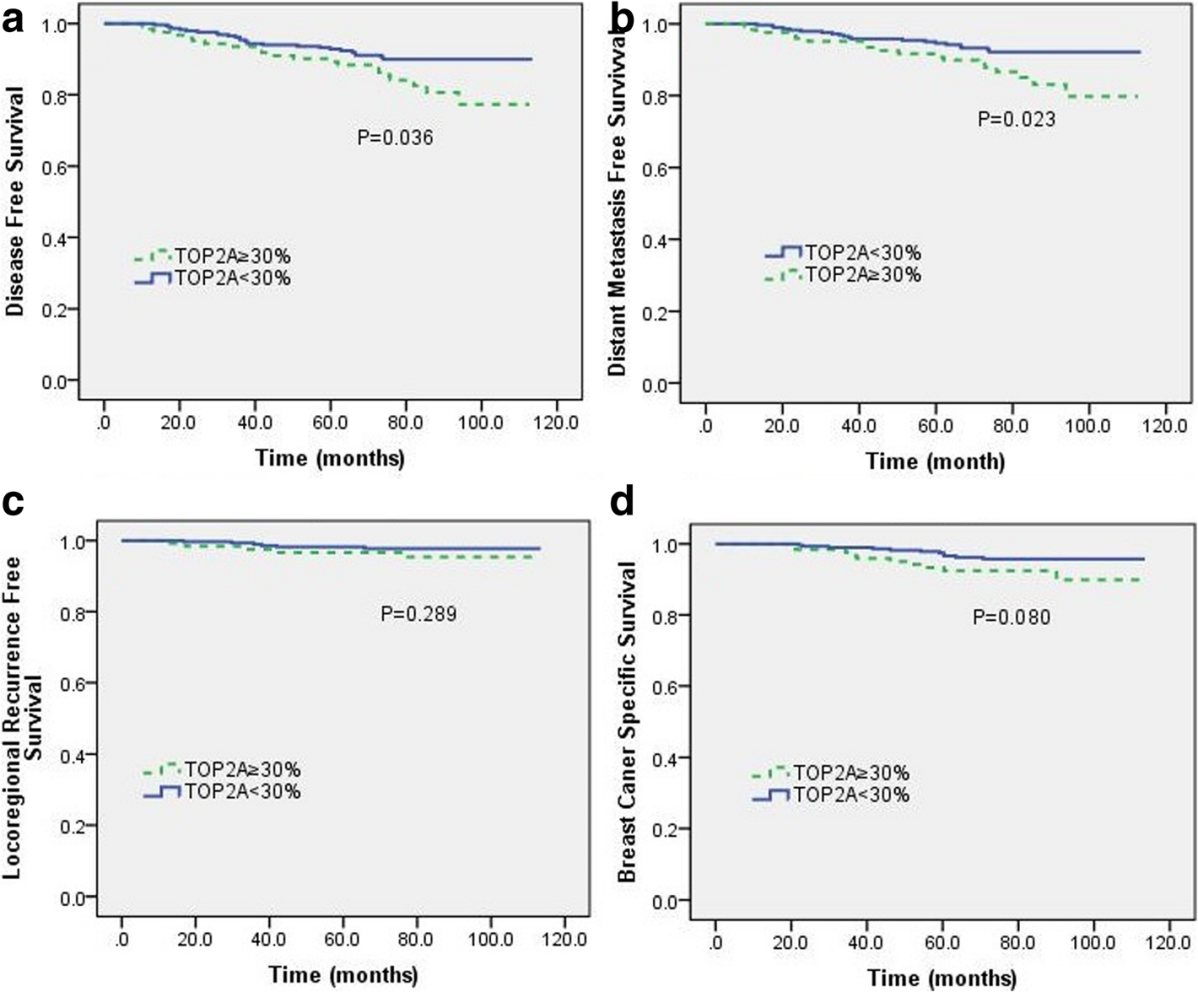
Kaplan-Meier survival analysis based on TOP2A expression. **a** disease-free survival (DFS); (**b**) distant metastasis-free survival (DMFS); (**c**) locoregional recurrent-free survival (LRFS); (**d**) breast cancer specific survival (BCSS)

Survival analysis showed that patients with high TOP2A expression showed significantly worse DFS and DMFS, but the difference was more pronounced after 5 years follow-up. The 5-year DFS and DMFS in TOP2A high and low group were 89.2% versus 92.8%, and 90.8% versus 94.6%. The 8-year DFS and DMFS in TOP2A high and low group were 77.4% versus 90.0%, and 83.1% versus 92.2%, respectively. No association between TOP2A expression and LRFS was observed. Patients with high TOP2A expression also showed a substantially worse BCSS, but the difference had no statistical significance. (Fig. 2).

The influence of TOP2A protein expression on DMFS was further stratified by pathologic stage, luminal subtypes, and adjuvant chemotherapy to better identify those patients who were really at high risk of distant metastasis. As a result, we found the unfavourable impact of TOP2A overexpression on DMFS was statistically significant in stage II disease (*P* = 0.025), luminal B patients (*P* = 0.046), and patients treated with adjuvant endocrine therapy alone without chemotherapy (*P* = 0.014) (Fig.. 3).

**Fig. 3:**
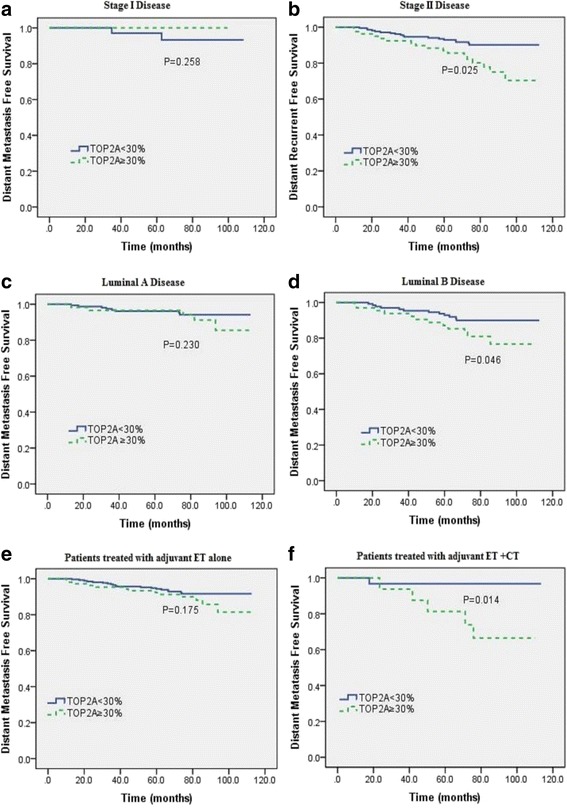
Subgroup survival analysis according to pathologic stage (**a**, **b**), molecular subtypes (**c**, **d**), and adjuvant therapy (**e**, **f**). Abbreviations: CT: chemotherapy; ET: endocrine therapy

Univariate and multivariate survival analysis revealed TOP2A overexpression was an independent fact for worse DMFS (*P* = 0.011). Other variables predicted poorer DMFS included young age (<=40 years old), high pathologic T stage (T3), lymph node metastasis (N1 disease), and high Ki67 index (≥20%). (Table 3).

**Table 3:** Univariate and multivariate analysis of variables correlated with metastasis-free survival

Variable	Univariate	Multivariate	
	*P*-value	HR (95% CI)	*P*-value
Age	0.007		0.027
≤ 40 vs. > 40,< 65		3.936(1.291–11.998)	0.016
> =65 vs. > 40,< 65		1.900(0.947–3.815)	0.071
Menopausal status	0.779		
Premenopausal vs. Postmenopausal			
Breast surgery	0.433		
Lumpectomy vs. Mastectomy			
PT	0.000		
T2 vs. T1		1.118(0.531–2.354)	0.769
T3 vs. T1		16.599(3.864–71.637)	0.000
PN	0.001	3.512(1.707–7.225)	0.001
N1 vs. N0			
histologic subtype	0.648		
Ductal vs. Lobular/orther			
Grade	0.079		
3 vs. 1–2			
Ki67	0.033	2.079(1.056–4.093)	0.034
≥ 20% vs. < 20%			
Hormone receptor expression	0.060		
ER(+) PR(−)/ER(−) PR(+) vs. ER(+) PR(+)			
Adjuvant CT	0.379		
Yes vs. No			
Adjuvant ET	0.615		
Yes vs. No			
Adjuvant RT	0.015	1.092(0.481–2.479)	0.834
Yes vs. No			
TOP2A	0.023	2.414(1.228–4.746)	0.011
High vs. Low			

*HR* Indicates hazard ratio, *CI* Confidence interval

*Abbreviations: PT* Pathologic T stage, *PN* Pathologic N stage

*CT* Chemotherapy, *ET* Endocrine therapy, *RT* Radiation

## Discussion

The current study demonstrated that TOP2A protein overexpression was associated with worse DFS, especially shorter DMFS in stage I-II luminal breast cancer. Moreover, the prognostic effect of TOP2A overexpression seemed to be time dependent with strong difference in years 5–8 after diagnosis. To our knowledge, this is the first study which purely focuses on a relatively large number of early-stage luminal breast cancer patients and shows the prognostic significance of TOP2A protein expression for late recurrence.

TOP2A is not a new marker for breast cancer. It has been well recognized as the molecular target of anthracyclines. Therefore, the majority of studies concerning TOP2A mainly focus on its potential predictive value for anthracyclines efficacy. However, conflicting results have been drawn [23, 24]. One reason might be due to different methods used to detect TOP2A status in different studies. Some studies detected TOP2A gene amplification by FISH, whereas others measured TOP2A protein expression by IHC. Poor agreement between these two methods has been observed [24, 25]. Several recent studies evaluated TOP2A mRNA expression via PCR or gene microarrays analysis and showed a quite good correlation with TOP2A protein expression [26, 27]. On the other hand, TOP2A protein is a proliferation marker which can be up-regulated by proliferative signals independently of its gene amplification [16, 28], thus could probably explain the poor correlation between TOP 2A protein expression and gene amplification. Since TOP2A protein represents the ultimate expression of TOP2A as well as tumor cell proliferation, therefore, detection of the protein may be better correlated with the tumor biology and predict the clinical outcome more precisely than genetic analysis.

Currently, IHC is the most commonly used and the easiest method to detect TOP2A protein expression. However, no standard antibodies, staining procedure, and scoring system have been recommended. Various cut-off values such as 5% [29], 10% [30], 15% [31], 20% [26], and 30% [32] have been applied in different studies. Other studies took both the staining intensity and percentage of positive cells into consideration to defined TOP2A status [33]. In the current study, we observed a strong positive correlation between nuclear TOP2A staining intensity and the percentage of positive cells. We tried all the different cut-off points mentioned about. As a result, a cut-off point of 30% was selected due to the best association with distant metastasis. According to the definition, 29.3% patients showed TOP2A overexpression.

In consistent with previous studies [30, 31, 34], we found TOP2A protein was significantly associated with high tumor grade and Ki67 index, suggesting that tumors with high level of TOP2A expression were more aggressive. No association between TOP2A expression and age, menstrual status, tumor size, lymph node status, pathological type were observed.

The prognostic effect of TOP2A protein expression on breast cancers is still in debates. Some studies showed that TOP2A overexpression was associated with poor outcome [19, 35]; others failed to demonstrate such an association [36]. One explanation might be the lack of standard procedure and definition of TOP2A protein by IHC as mentioned above. Another probable reason may be due to different prognostic impact of TOP2A protein on different subtypes of breast cancer. Peter Fritz and his colleagues [19] enrolled 225 un-subtyped operable breast cancer patients, and found TOP2A predicted prognosis only in hormone receptor positive disease. Rody et al. [20] revealed a significant prognostic value of TOP2A mRNA in ER positive breast cancer by analyzing microarray data of 1681 patients. Another most recent study evaluated an even larger database of TOP2A mRNA in 4142 breast cancer patients, and reported again that high TOP2A mRNA expression was only significantly associated with poor prognosis in luminal breast cancer [21]. In consistent with these three studies, the current study demonstrated that high TOP2A protein expression was a worse prognostic factor in early stage luminal breast cancer. Patients with high TOP2A expression showed a significant higher rate of distant metastasis compared with that for patients with low TOP2A expression. The most interesting thing is that the prognosis effect of TOP2A seemed be time dependent with strong difference in years 5–8 after diagnosis, suggesting TOP2A protein overexpression might be a potential predictor of late recurrence. High TOP2A expression was also associated with a trend of higher breast cancer specific death, but the differences did not reach statistical significance. This result is easy to understand. Luminal breast cancer generally shows long survival even after recurrence, therefore, might need longer follow-up to observe the difference. Also subsequent treatment for recurrent disease would influence the survival. Multivariate analysis revealed the prognostic impact of TOP2A protein was independent of Ki67 index, as well as other clinicopathological factors including age, pathologic T and N stage. Subgroup analysis showed the unfavourable impact of high TOP2A expression was more significant in stage II and luminal B patients. Probably due to these subgroup of patients had a relatively poor outcome and a higher incidence of distant metastases. It is noteworthy that we found high TOP2A level was associated with a significantly poorer DMFS in patients treated with adjuvant endocrine therapy alone. For patients received additional adjuvant chemotherapy, although DMFS was still worse in TOP2A high group than in TOP2A low group, the difference did not reach statistically significance. This finding suggests at least two things: one is that TOP2A is a prognostic factor for early stage luminal breast cancer treated with adjuvant endocrine therapy alone; another is that TOP2A might be a predictive factor for benefit from adjuvant chemotherapy.

The main limitation of current study is the retrospective analysis. Although the sample size is relatively large, the really good prognosis for this cohort of patients, and the low number of total events will to some extent limit the statistical power. Moreover, the number of patients in our datasets who were given adjuvant endocrine therapy alone without chemotherapy is too small, and the heterogeneity of adjuvant chemotherapy regimens should also be acknowledged as limiting factors for this study.

## Conclusion

The current study for the first time demonstrated the worse and time dependent prognostic impact of TOP2A overexpression in early stage luminal breast cancer patients, suggesting the potential value of TOP2A as a predictor of late recurrence for this subtype of breast cancer. Further large-scaled, prospective studies with standardized method of measuring TOP2A are required to overcome the limitations of current study and confirm the utility of TOP2A protein in luminal breast cancer.

## Abbreviations

AJCC: American Joint Committee on Cancer stage system
BCSS: Breast cancer specific survival
DFS: Disease-free survival
DMFS: Distant metastasis-free survival
ER: Estrogen receptor
GEP: Gene expression profiling
HER2: Human epidermal growth factor receptor 2
IHC: IMMUNOHISTOCHEMISTRY
LRFS: Locoregional recurrence-free survival
OS: Overall survival
PgR: Progesterone receptor
RT-qPCR: Quantitative reverse-transcriptase polymerase chain reaction
TOP2A: Topoisomerase II alpha
